# HEALTH AND AGING AMONG LATIN AMERICAN OLDER ADULTS: FINDINGS FROM STUDIES IN BRAZIL, CHILE, COLOMBIA, AND MEXICO

**DOI:** 10.1093/geroni/igad104.1244

**Published:** 2023-12-21

**Authors:** Margarita Maria Osuna, Jennifer Ailshire

## Abstract

Aging in Latin America is occurring rapidly, in a context of high levels of poverty, low education and limited knowledge on the health conditions of older adults. This symposium is focused on heath-disparities found in some of Latin America’s largest middle-income countries, Mexico, Brazil, Chile, and Colombia. The symposium explores various health-related topics associated with aging, that may have significant implications for longevity, overall health, and disease risk. The papers in this symposium examine a variety of health-related dimensions and disparities among older Latinos that include major chronic health conditions, such as diabetes, hypertension, cardiovascular diseases and cognition. Using data from the Brazilian Longitudinal Study of Aging, Farina examines the relationship between childhood conditions and older adult health. Using the Mexican Health and Aging Study (MHAS) and the Health and Retirement study (HRS), Cantu compares cognitive measures and their association with gender in Mexico and the US. Using mortality data, Sandoval studies how healthy life expectancy varies among the largest indigenous group in Chile compared to non-indigenous populations. Lastly, Osuna uses data from the Colombian Survey of Health, Well-Being, and Aging to examine how diseased life expectancy varies from self-reported data compared to measured data among older Colombians. Results underline what populations in Latin American have increased risk of experiencing poorer health outcomes and disparities that may exist. The findings highlight the importance of understanding the conditions under which Latin American older adults are aging and the implications this can have in the future.

